# Co‐Oxidative Transformation of Piperine to Piperonal and 3,4‐Methylenedioxycinnamaldehyde by a Lipoxygenase from *Pleurotus sapidus*

**DOI:** 10.1002/cbic.202100183

**Published:** 2021-06-09

**Authors:** Nina‐Katharina Krahe, Ralf G. Berger, Lukas Kahlert, Franziska Ersoy

**Affiliations:** ^1^ Institut für Lebensmittelchemie Gottfried Wilhelm Leibniz Universität Hannover Callinstr. 5 30167 Hannover Germany; ^2^ Present address: Institut für Organische Chemie und Biomolekulares Wirkstoffzentrum Gottfried Wilhelm Leibniz Universität Hannover Schneiderberg 38 30167 Hannover Germany

**Keywords:** biotransformations, cleavage reactions, co-oxidation, lipoxygenase, piperonal

## Abstract

The valuable aroma compound piperonal with its vanilla‐like olfactory properties is of high interest for the fragrance and flavor industry. A lipoxygenase (LOX_Psa_1) of the basidiomycete *Pleurotus sapidus* was identified to convert piperine, the abundant pungent principle of black pepper (*Piper nigrum*), to piperonal and a second volatile product, 3,4‐methylenedioxycinnamaldehyde, with a vanilla‐like odor through an alkene cleavage. The reaction principle was co‐oxidation, as proven by its dependence on the presence of linoleic or *α*‐linolenic acid, common substrates of lipoxygenases. Optimization of the reaction conditions (substrate concentrations, reaction temperature and time) led to a 24‐fold and 15‐fold increase of the piperonal and 3,4‐methylenedioxycinnamaldehyde concentration using the recombinant enzyme. Monokaryotic strains showed different concentrations of and ratios between the two reaction products.

## Graphical abstract

**Biotransformation of piperine to piperonal and 3,4‐methylenedioxycinnamaldehyde**: A lipoxygenase from *Pleurotus sapidus* (LOX_Psa_1) was identified, which generated the highly valuable, vanilla‐like aroma compound piperonal through co‐oxidation of piperine, the pungent principle of black pepper, in the presence of polyunsaturated fatty acids. In addition, 3,4‐methylenedioxycinnamaldehyde, which also presents a vanilla‐like odor, was produced as second product.

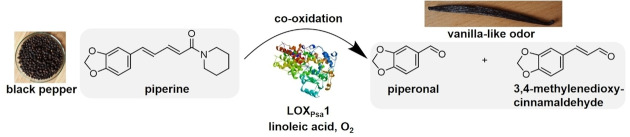

Piperonal (3,4‐methylenedioxybenzaldehyde or Heliotropin) has a sweet‐flowery, vanilla‐like odor that is of high interest for the fragrance and flavor industry.^[1]^ It is present in low amounts in different plants, such as violet flower, robinia, meadowsweet, and vanilla. To meet the high demand, it is traditionally produced by chemical synthesis.^[1]^ However, the rising popularity of natural products and sustainable production processes require alternative strategies, such as biocatalysis.^[2]^ Bioconversion of isosafrole, piperonyl alcohol, and piperonylic acid to piperonal has been shown by different enzymes or bacterial and fungal strains.[^1^, ^3^, ^4^, ^5^] However, some of these use non‐natural substrates, thus resulting in non‐natural piperonal.^[2]^ Even though isosafrole is a natural starter, it suffers from its limited natural occurrence, high prices, and legislative restrictions as it works as precursor of 3,4‐methylenedioxy‐N‐methylamphtamine (ecstasy).[^6^, ^7^]

Lipoxygenases (EC: 1.13.11.12; LOX) are non‐heme, mostly iron containing dioxygenases and ubiquitously present in eukaryotic organisms. They catalyze the regio‐ and stereospecific dioxygenation of (*cis*)‐polyunsaturated fatty acids (PUFAs) to their corresponding unsaturated fatty acid hydroperoxides by a radical mechanism.[^8^, ^9^] Co‐oxidation of different unsaturated compounds by the enzyme's reactions products first became obvious due to the visually observed bleaching of pigments.[^10^, ^11^] Investigations using antioxidants confirmed the radical character of the co‐oxidation mechanisms.^[12]^ However, the mechanisms behind these effects are not yet fully understood.^[9]^

Herein, we present the bioconversion of piperine, the main alkaloid and pungent aroma principle of black pepper, to natural piperonal and an additional aroma compound, 3,4‐methylenedioxycinnamaldehyde, by *Pleurotus sapidus* (Scheme 1). A lipoxygenase, which was known to convert (+)‐valencene to the grapefruit aroma (+)‐nootkatone,[^13^, ^14^] was identified as responsible for the reaction through co‐oxidation. To the best of our knowledge, this is the first study using piperine for the biocatalytic generation of natural piperonal and the first report describing cleavage of aryl alkenes by a lipoxygenase activity.

**Scheme 1:**
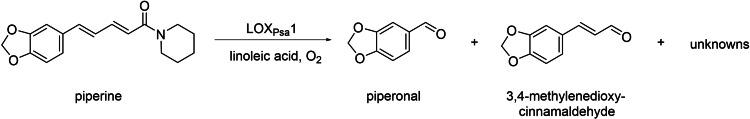
Co‐oxidation of piperine to piperonal and 3,4‐methylenedioxycinnamaldehyde during linoleic acid oxidation by LOX_Psa_1 from *P. sapidus*.

An alkene cleavage activity of the mycelium of the basidiomycete *P. sapidus* degraded 98 % of piperine and generated 44.2±0.1 μM piperonal (6.4 % molar yield) (Figures S1 and S2). Additionally, 51.8±1.2 μM 3,4‐methylenedioxycinnamaldehyde ((2*E*)‐3‐(1,3‐benzodioxol‐5‐yl)‐2‐propenal; 7.3 % molar yield) resulted from the competing cleavage of the second double bond of piperine. Olfactometric analysis of 3,4‐methylenedioxycinnamaldehyde revealed a sweet odor, reminding of vanilla, which was in line with speculations of Kollmannsberger *et al*.^[15]^ Thus, a second potentially interesting aroma compound was generated. Further volatile products were not detected (Figure S1), although the significantly higher degradation of piperine in comparison to the product yield indicated the formation of further by‐products. Potentially, consecutive reactions, such as polymerizations, resulted in non‐volatile products.

To identify the enzyme catalyzing the biotransformation, semi‐purification from the soluble part of the rehydrated mycelium (crude extract) was tested. However, no piperine cleaving activity was found. To improve enzyme stability and solubility, different agents were tested (Figure S3). Dithiothreitol and to a lesser extend glutathione had a significant stabilizing effect on the piperine cleaving activity. Thus, the enzyme was sensitive to sulfhydryl oxidation, which results in disulfide bond formation and most likely in conformational changes of the protein structure, which negatively affected the activity.^[16]^

An activity loss during purification can also result from the loss of cofactors or co‐substrates, as H_2_O_2_‐ and/or manganese‐dependency is known for other alkene cleaving basidiomycetous enzymes.[^3^, ^17^] The loss of the LMMF (low molecular mass fraction) decreased the bioconversion (Figure 1a), thus confirming its requirement for the piperine cleaving enzyme. Supplementation of H_2_O_2_ and Mn^2+^ (MnSO_4_) showed that addition of Mn^2+^ increased the piperonal generation (Figure 1b).

**Figure 1:**
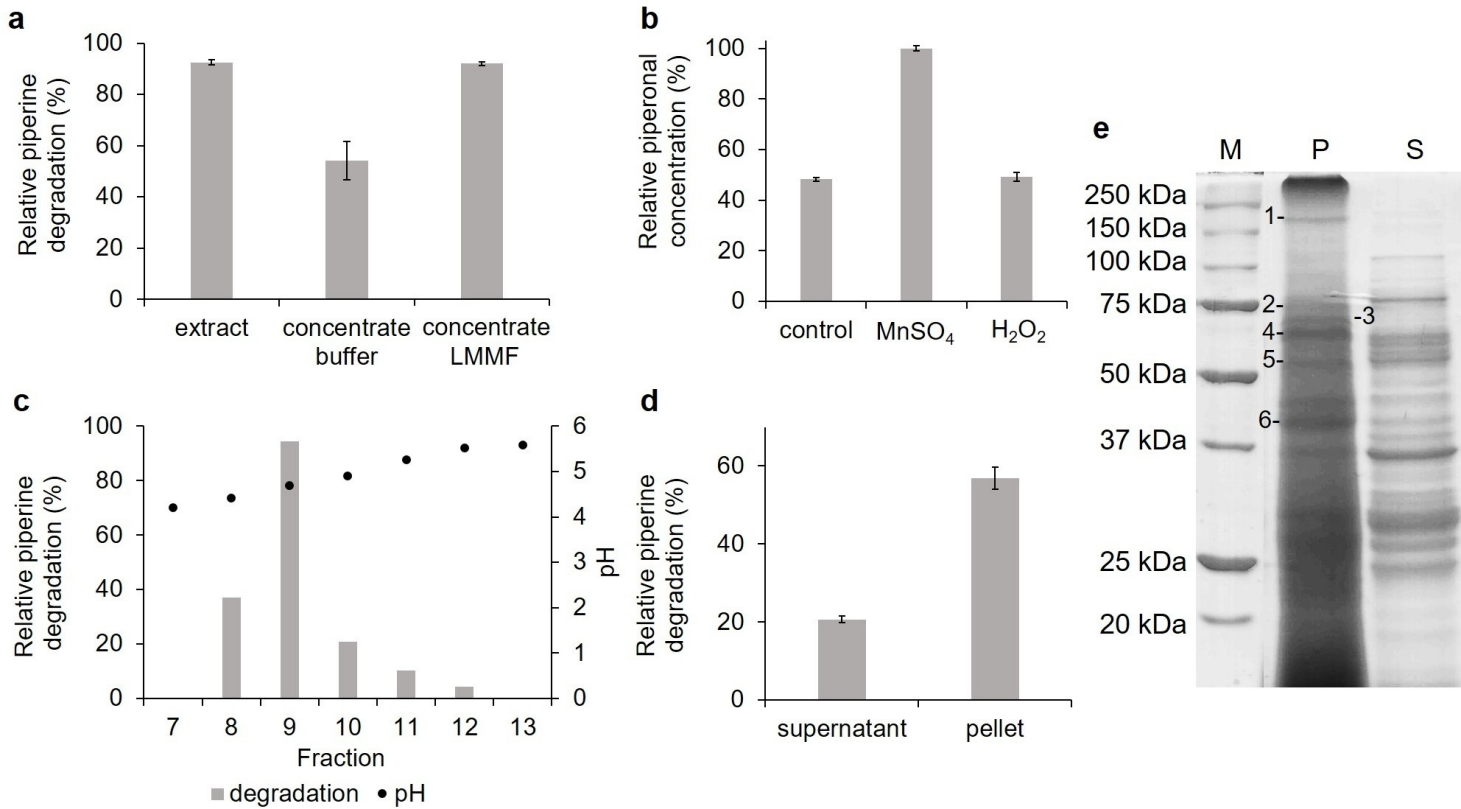
Activity analysis and semi‐purification of the piperine cleaving enzyme. **a**) Influence of the low molecular mass fraction (LMMF) on the piperine degradation by *P. sapidus*. Extract: crude extract in buffer (25 mM Tris‐HCl, pH 8.0). The extract was concentrated by ultrafiltration (3 kDa cut‐off) and filled up to the starting volume with buffer or LMMF. **b**) Piperonal concentration after biotransformation in the absence (control) or presence of 1 mM MnSO_4_ or 100 μM H_2_O_2_ using the crude extract. Concentrations were calculated relative to the highest piperonal concentration. **c**) Analysis of the preparative IEF fractions. **d**) Piperine degradation by the soluble (supernatant) and insoluble part (pellet) of fraction 8 to 10 of the preparative IEF. The pellet was re‐suspended in buffer for analysis (same volume as before centrifugation). **e**) SDS‐PAGE of the soluble and insoluble part of fraction 9 after preparative IEF. M: molecular mass marker, P: pellet, S: supernatant. The bands excised for protein sequencing are marked 1 to 9. Piperine degradation (a, c, d) was calculated relative to the starting piperine concentration. All experiments were performed in the presence of 1 mM piperine at pH 4.5 and RT for 16 h.

Semi‐purification of the desired enzyme was performed *via* preparative isoelectric focusing (IEF) after addition of dithiothreitol, thus avoiding washing steps, which would have resulted in a high loss of the LMMF. The biotransformation reaction with the collected fractions was performed in the presence of Mn^2+^ and additional LMMF. Piperine cleavage was observed for fractions collected at pH 4.4 to 5.5 with the highest activity in fraction 9 (pH 4.7) (Figure 1c). A white protein precipitate in the active fractions 8 to 10 contained most of the activity (Figure 1d). Precipitation at the isoelectric point is well known for proteins.^[18]^

SDS‐PAGE analysis revealed a multitude of protein bands in the insoluble part of fraction 9 (Figure 1e). However, further purification would have been challenging due to the LMMF‐dependency, a low fraction volume (<500 μL), and the disability to re‐dissolve the precipitated piperine cleaving enzyme completely. Thus, the most dominating protein bands, which were not or less present in the other preparative IEF fractions (data not shown) and the soluble part of fraction 9, were excised and digested using trypsin for electrospray ionization tandem mass spectrometry (Figure 1e). Homology searches of the identified peptides using the NCBI database and the mascot search engine (Matrix Science, London, UK) revealed two enzymes potentially responsible for the piperine biotransformation (Table S1): A lipoxygenase (LOX_Psa_1)^[14]^ and a dye‐decolorizing peroxidase (DyP; PsaPOX).[^17^, ^19^] The latter is known to cleave different aryl alkenes but not piperine.^[17]^ Thus, LOX_Psa_1 was further investigated.

Recombinant LOX_Psa_1 was produced in *E. coli*, purified by Ni‐NTA affinity chromatography^[13]^ (Figure S4; specific activity for linoleic acid: 667 nkat/mg, 40 U/mg), and used for biotransformation experiments (Figure 2). LOX_Psa_1 (100 nkat/mL, 6 U/mL;) converted piperine into piperonal and 3,4‐methylenedioxycinnamaldehyde (ratio ∼0.5 : 1) in the presence of linoleic acid, a known substrate of LOX_Psa_1 (Figures 2a and S5).^[14]^ No activity was observed without linoleic acid. This indicated that piperine was not a direct substrate, but most likely cleaved by co‐oxidation during linoleic acid oxidation (Scheme 1). Recent work suggested that various members of the catalytic cycle of lipoxygenases might interact with unsaturated substrates in co‐oxidation reactions.^[8]^ In the presented case, the initial linoleic acid hydroperoxide radicals may abstract hydrogens from the unsaturated bridge of piperine paving the way for an autoxidative insertion of an oxygen molecule. As a stable dioxene‐ or hydroperoxo‐intermediate was not found, the exact mechanistic route remains obscure. In the mycelium and crude extract, fungal PUFAs most likely initialized the co‐oxidation process as substrates, which would well explain the activity loss during the initial purification attempts and the LMMF‐dependency (Figure 1a). In contrast to the results for the crude extract, addition of Mn^2+^ had no influence on the biotransformation yield of LOX_Psa_1 (Figures 1b and 2a). A second, Mn^2+^‐dependent enzyme may participate in the piperine conversion. This remains to be elucidated in a follow‐up study.

**Figure 2:**
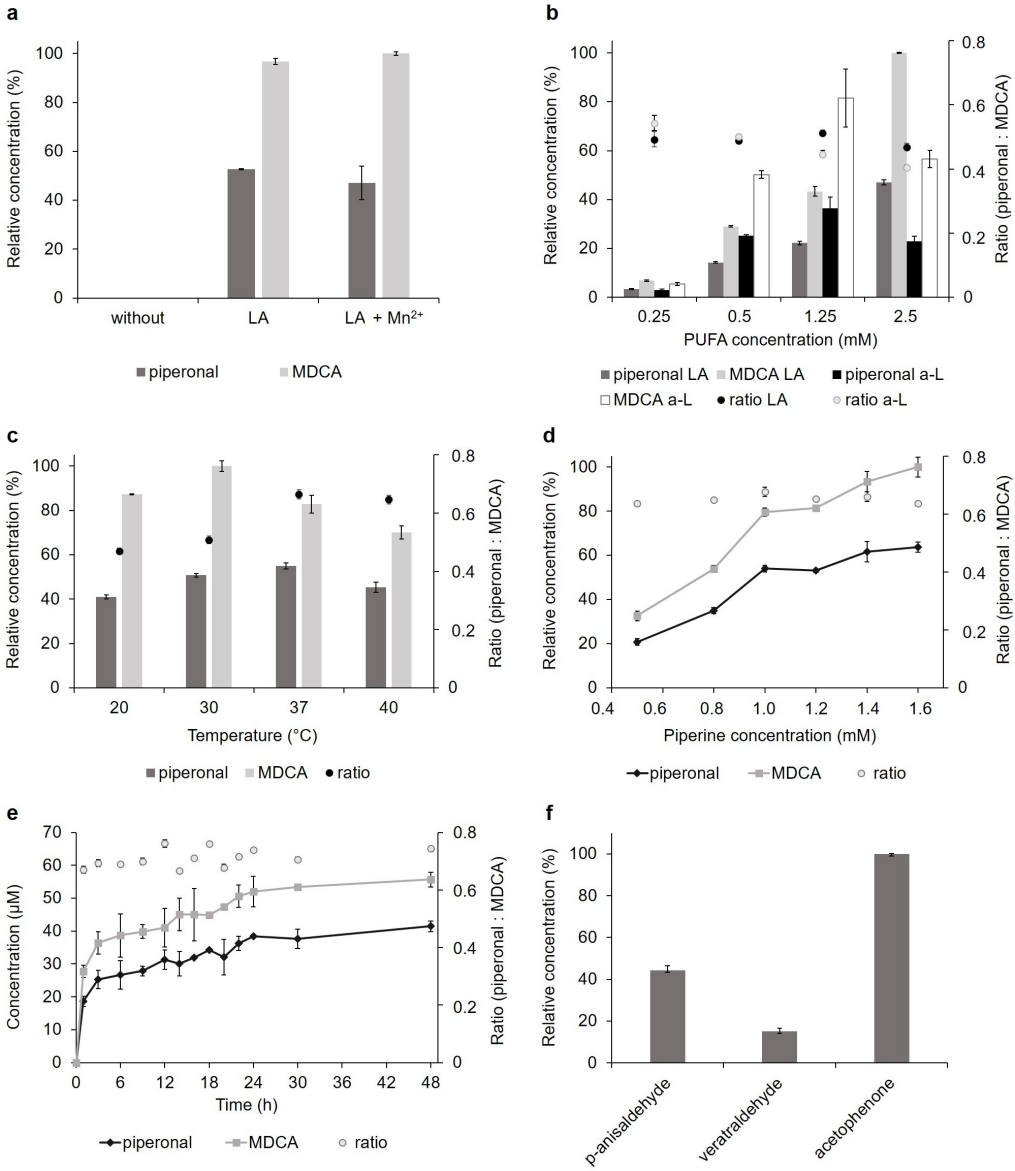
Biotransformation of piperine by the recombinant LOX_Psa_1 (100 nkat/mL, 6 U/mL). **a**) Biotransformation of 1 mM piperine in the absence or presence of 0.25 mM linoleic acid (LA) and 1 mM Mn^2+^ (RT). **b**) Influence of the LA and linolenic acid (a‐L) concentration (1 mM piperine, RT). **c**) Temperature optimum (2.5 mM LA, 1 mM piperine). **d**) Influence of the piperine concentration (2.5 mM LA, 37 °C). **e**) Reaction kinetic of the piperonal and 3,4‐methylenedioxycinnamaldehyde formation (2.5 mM LA, 1.6 mM piperine, 37 °C). **f**) Product concentration after conversion of *trans*‐anethole to *p*‐anisaldehyde, (*E*)‐methyl isoeugenol to veratraldehyde, and *α*‐methylstyrene (all 6.7 mM) to acetophenone (2.5 mM LA, 37 °C). Relative product concentrations were defined as relative to the highest product concentration obtained in each experiment. All experiments were performed at pH 7 for 16 h (exemption: kinetic in e). MDCA: 3,4‐methylenedioxycinnamaldehyde.

To increase the biotransformation yield, different PUFAs and concentrations were examined as well as the influence of pH, temperature, piperine concentration, and incubation time (Figures 2b–e and S6). Biotransformation experiments with linoleic and *α*‐linolenic acid showed that the product concentration increased significantly with rising PUFA concentrations (up to 17.5‐fold; exemption: 2.5 mM *α*‐linolenic acid) (Figure 2b). The PUFA concentration was the parameter with the highest effect on the biotransformation yield. These findings support the co‐oxidative character of the piperine cleavage reaction. Linoleic acid at the highest concentration (2.5 mM) achieved the highest piperonal (25 μM) and 3,4‐methylenedixycinnamaldehyde concentrations (53 μM) and was thus used for all subsequent assays.

Analysis of the piperine biotransformation revealed a pH optimum of 7 (Figure S6) and a temperature optimum of 30 °C (overall product concentration, Figure 2c). These results agreed with the optima reported for the linoleic acid oxidation by LOX_Psa_1.^[14]^ However, the product ratio of piperonal to 3,4‐methylenedioxycinnamaldehyde increased from 0.5 to 0.65 at 37 °C (Figure 2c). This most likely resulted from thermodynamic effects,^[20]^ which disfavor the cleavage of the second double bond and hence 3,4‐methylenedioxycinnamaldehyde formation at higher temperatures. As piperonal is the more valuable cleavage product, 37 °C was considered as optimal for piperonal synthesis and used for the following experiments. Temperature was the only parameter that effected the product‐ratio (Figures 2b–e and S6).

Additional experiments showed a linear increase in product concentration with rising piperine concentrations (Figure 2d, coefficient of determination R^2^≥0.90). Concentrations higher than 1.6 mM piperine were not investigated due to the lack of solubility. An increase of the incubation time to 48 h resulted in the highest overall piperonal (41 μM) and 3,4‐methylenedioxycinnamaldehyde concentrations (56 μM) (Figure 2e). Thus, improving the reaction conditions (linoleic acid and piperine concentration, reaction temperature and time) achieved a 24‐ and 15‐fold increase of the piperonal and 3,4‐methylenedioxycinnamaldehyde concentrations, respectively.

During the first three hours, over 60 % of the maximal product concentration was obtained (Figure 2e). The following decrease of the biotransformation rate was most likely the result of a linoleic acid limitation, as it was completely degraded after 16 h (Figure S5). Higher linoleic acid concentrations of a fed‐batch regime may be applied. In addition, higher enzyme concentrations may be used, as they led to increased product formation (Figure S7).

LOX_Psa_1 was further examined for bioconversion of other alkenes. The aryl alkenes *trans*‐anethole, (*E*)‐methyl isoeugenol, and *α*‐methylstyrene were converted to the expected olfactants *p*‐anisaldehyde, veratraldehyde, and acetophenone, respectively (Figure 2f and Scheme S1). The highest product concentration was identified for *α*‐methylstyrene followed by *trans*‐anethole (about two‐fold lower) and (*E*)‐methyl isoeugenol (about six‐fold lower).

In summary, the biocatalytic generation of piperonal using piperine as substrate was achieved by a co‐oxidation reaction catalyzed by LOX_Psa_1 in the presence of linoleic acid. In addition, a second aroma compound, 3,4‐methylenedioxycinnamaldehyde, was generated, which also offered a vanilla‐like odor. Separation of both aldehydes may be achieved by adsorption to zeolithes as shown, for example, for limonene and carvone.^[21]^ Alternatively, a combined application could be envisaged due to the similar odor attributes. Although the improved reaction conditions increased the product concentrations, further optimization is needed. Besides higher linoleic acid concentrations, monokaryotic daughter‐strains of *P. sapidus* are an option, as they showed higher LOX activities^[22]^ and higher product concentrations after piperine transformation (Figure S8). In addition, some of the daughter strains favored the formation of piperonal over 3,4‐methylenedioxycinnamaldehyde. As LOX_Psa_1 converted further aryl alkenes to their respective odor‐active aldehydes, it showed potential as biocatalyst for aroma production. However, further optimization is needed to improve product concentrations for a potential industrial application.

## Experimental Section

Experimental details are given in the Supporting Information.

## Conflict of interest

The authors declare no conflict of interest.

## Supporting information

As a service to our authors and readers, this journal provides supporting information supplied by the authors. Such materials are peer reviewed and may be re‐organized for online delivery, but are not copy‐edited or typeset. Technical support issues arising from supporting information (other than missing files) should be addressed to the authors.

Supporting Information
