# Supplementary material for: Co‐Oxidative Transformation of Piperine to Piperonal and 3,4‐Methylenedioxycinnamaldehyde by a Lipoxygenase from Pleurotus sapidus
Source: Chembiochem. 2021 Jun 9;22(19):2857–61. doi: 10.1002/cbic.202100183 (PMC8518924; doi:10.1002/cbic.202100183)
Supplement: Supplementary file 1 — Supporting Information [file CBIC-22-2857-s001.pdf]

ChemBioChem

Supporting Information

Co-Oxidative Transformation of Piperine to Piperonal and 3,4-Methylenedioxycinnamaldehyde by a Lipoxygenase from *Pleurotus sapidus*

Nina-Katharina Krahe,* Ralf G. Berger, Lukas Kahlert, and Franziska Ersoy

Author Contributions

N.-K.K. Conceptualization:Equal; Data curation:Lead; Formal analysis:Lead; Investigation:Lead; Methodology:Lead; Validation:Equal; Visualization:Lead; Writing – original draft:Lead
R.B. Conceptualization:Equal; Funding acquisition:Lead; Project administration:Lead; Resources:Lead; Validation:Equal; Visualization:Supporting; Writing – review & editing:Equal
L.K. Formal analysis:Supporting; Investigation:Supporting; Methodology:Supporting; Validation:Supporting; Writing – review & editing:Equal
F.E. Conceptualization:Equal; Supervision:Lead; Validation:Equal; Writing – review & editing:Equal

Supporting Information

Table of Contents

1. Materials and Methods 2

1.1 Chemicals and Materials 2

1.2 Cultivation of *P. sapidus* 2

1.3 Purification strategy 2

1.4 Heterologous expression and purification of $LOX_{Psa}1$ 3

1.5 Biotransformation 3

1.5.1 *P. sapidus* mycelium 3

1.5.2 Recombinant $LOX_{Psa}1$ 4

1.5.3 Biotransformation of other aryl alkenes 4

1.6 HPLC analysis for piperine quantification 4

1.7 GC analysis 5

1.7.1 Quantification of piperonal and 3,4-methylenedioxycinnamaldehyde after stir bar sorptive extraction 5

1.7.2 Quantification of piperonal and 3,4-methylenedioxycinnamaldehyde after liquid extraction 6

1.7.3 Quantification of *p*-anisaldehyde, veratraldehyde, and acetophenone 6

2. Results 7

Figure S1. TDS-GC-FID chromatogram of the bioconversion of piperine with lyophilized mycelium of *P. sapidus* after 16 h of incubation. 7

Figure S2. MS spectra resulting from GC-MS analysis that were used for the allocation of the substrate and products to the respective peaks in the TDS-GC-FID chromatogram (Figure S1). 8

Figure S3. Storage stability of the piperine cleaving enzyme in the soluble fraction after rehydration of the *P. sapidus* mycelium. 9

Table S1. Best hits resulting from the protein sequencing of protein band 1 to 6 from the SDS-PAGE (Figure 1e) 10

Figure S4. SDS-PAGE analysis of the purified recombinant $LOX_{Psa}1$. 11

Figure S5. TDS-GC-FID chromatogram of the bioconversion of piperine with $LOX_{Psa}1$ after 16 h of incubation. 12

Figure S6. pH optimum of the piperine biotransformation using 100 nkat/mL (6 U/mL) $LOX_{Psa}1$ in the presence of 2.5 mM linoleic acid and 1 mM piperine at RT after 16 h. 13

Figure S7. Piperine biotransformation with different $LOX_{Psa}1$ activities in the presence of 2.5 mM linoleic acid and 1 mM piperine at pH 7 and RT after 16 h. 13

Scheme S1. Bioconversion of **a**) *trans*-anethole to *p*-anisaldehyde, **b**) (*E*)-methyl isoeugenol to veratraldehyde, and **c**) *α*-methylstyrene to acetophenone by $LOX_{Psa}1$. 14

Figure S8. Piperine biotransformation with different *P. sapidus* strains in the presence of 2.5 mM linoleic acid and 1 mM piperine at pH 7 and RT after 16 h. 14

3. References 14

1. Materials and Methods

1.1 Chemicals and Materials

Chemicals were obtained from Sigma Aldrich (Seelze, Germany), Carl Roth (Karlsruhe, Germany), or Merck (Darmstadt, Germany) in *p. a.* quality, if not stated otherwise. A piperonal standard was synthesized according to Gallagher *et al.* using aqueous $KMnO_4$.[1]

1.2 Cultivation of *P. sapidus*

P. sapidus (Deutsche Sammlung von Mikroorganismen und Zellkulturen GmbH, DSMZ, strain no. 2866 and nine monokaryotic daughter strains[2]) was pre-grown on 1.5% (w/v) agar plates with standard nutrient liquid (SNL) medium and maintained at 4 °C until use.[3] For pre-cultivation, 1 cm^2 of grown agar was transferred to 100 mL SNL medium and treated with an Ultraturrax homogenizer (ART Prozess- & Labortechnik, Müllheim, Germany). The pre-cultures were incubated for 5 days at 150 rpm and 24 °C. Afterwards, 6.5 g wet biomass was used to inoculate 250 mL SNL. The main culture was incubated at 150 rpm and 24 °C. After six days, the mycelium was separated from the culture supernatant by centrifugation (5000× g, 4 °C, 15 min) and lyophilized as described elsewhere.[4] Afterwards, the lyophilisates were finely ground.

1.3 Purification strategy

25 g lyophilized mycelium was re-suspended in 600 mL 25 mM Tris-HCl, pH 8 in the presence of 2 mM dithiothreitol and extracted for 2 h at 4 °C in horizontal position in an orbital shaker (200 rpm). Insoluble components were removed by centrifugation (5000× g, 4 °C, 15 min) followed by filtration (PES filter, 0.45 µm, Merck). Additional 2 mM dithiothreitol was added to the cell free crude extract before the extract was concentrated (ten-times) by cross-flow filtration (10 kDa cut-off, Sartocon® Slice PESU Cassette, Sartorius, Göttingen, Germany). The concentrate was mixed with 2% Servalyt (pH 3–6; Serva, Heidelberg, Germany) and 2 mM dithiothreitol and transferred to the focusing chamber of a Rotofor cell (Bio-Rad, Munich, Germany). The chamber was cooled to 2 °C. Focusing was carried out at constant power of 12 W using 0.5 M ethanoleamine and 0.5 M acetic acid as anode and cathode buffers, respectively. After 4 h, 20 fractions were harvested. Each fraction was analyzed regarding its pH and its capability to convert piperine. SDS-PAGE analysis and peptide mass fingerprinting was performed as described elsewhere.[5,6] The obtained partial peptide sequences were used for similarity searches against the public databases NCBI using the mascot search engine (Matrix Science, London, UK).

1.4 Heterologous expression and purification of $LOX_{Psa}1$

The recombinant lipoxygenase ($LOX_{Psa}1$) was produced in *E. coli* BL21 DE3 Star and purified *via* Ni-NTA affinity chromatography as described elsewhere.[7,8] Protein concentrations were determined according to Bradford[9] using bovine serum albumin as standard. Lipoxygenase activities were determined photometrically by monitoring the absorbance change at 234 nm due to the oxidation of 0.25 mM linoleic acid to the respective conjugated hydroperoxydienes at pH 7 and 30 °C following the previously established procedure by Plagemann *et al.*[10]

1.5 Biotransformation

All experiments were performed as fourfold determination (one duplicate each for GC analysis and HPLC analysis) or in duplicates (only GC analysis). For experiments with *P. sapidus* mycelium or crude extract independent biological replicates were used. The data are shown in the main text and below are the average of the duplicate experiments with standard deviations shown as arrow bars.

For all experiments blanks were performed without *P. sapidus* mycelium or recombinant enzyme (chemical blank) or with heat inactivated mycelium or enzyme (4 h (mycelium) or 1 h (enzyme) at 95 °C, biological blank). The determined product concentrations for the blanks were subtracted from the concentrations yielded for the reaction with the active samples to calculate the enzymatically generated product concentration.

1.5.1 *P. sapidus* mycelium

Transformation of piperine was carried out in 4 mL gas tight glass vials in horizontal position at a shaking rate of 200 rpm for 16 h at RT in the absence of light. Reaction mixtures contained 1 mM piperine and 30 mg *P. sapidus* lyophilisate or 250 µL liquid sample (crude extract or preparative IEF fraction) buffered in sodium acetate (50 mM, pH 4.5) with or without addition of 1 mM $MnSO_4$ or 100 µM H_2O_2 in a total volume of 1 mL. For the analysis of the enzyme stability, cell free crude extract (see Supporting Information, section 1.3) was stored in the presence of a peptidase inhibitor mix (0.5 mM phenylmethylsulfonyl fluoride, 1.5 µM aprotinin, 50 µM bestatin, 10 µM pepstatin A), dithiothreitol (2 and 10 mM), glutathione (2 and 10 mM), Triton X-100 (0.01 and 0.04% (w/v)), Tween 20 (0.001 and 0.01% (w/v)), 3-[(3-cholamidopropyl)dimethylammonio]-1-propanesulfonate (CHAPS; 0.1 and 0.7% (w/v)), bovine serum albumine (BSA, 1% (w/v)), or glycerol (5% (w/v)) at 4 °C for 0 h to 7 d before biotransformation. For analysis of the influence of the low molecular mass fraction (LMMF) of the crude extract on the biotransformation reaction, the extract was concentrated (10-times) by ultrafiltration (3 kDa cut-off, polyethersulfone (PES), Sartorius) and refilled to the starting volume with Tris-HCl (25 mM, pH 8) or LMMF before bioconversion.

Analysis of the biotransformation products was performed *via* GC after stir bar sorptive extraction (see Supporting Information, Section 1.7.1).

1.5.2 Recombinant $LOX_{Psa}1$

The purified recombinant lipoxygenase (100 nkat/mL (6.0 U/mL), 0.15 mg/mL) was used for transformation of piperine as mentioned above to confirm alkene cleavage activity. Conversion of 1 mM piperine was performed in the presence or absence of linoleic or α-linolenic acid (0.25–2.5 mM) in sodium phosphate buffer (25 mM, pH 7) at RT for 16 h. In addition, supplementation of 1 mM $MnSO_4$ in the presence of 0.25 mM linoleic acid was tested. The pH optimum was determined using Britton-Robinson buffer[11] in a range of pH 5.0–9.0 in the presence of 2.5 mM linoleic acid at RT, while for analysis of the temperature optimum the bioconversion was performed at different temperatures (20–90 °C) in the presence of 2.5 mM linoleic acid at pH 7 (25 mM sodium phosphate buffer). The influence of the piperine concentration was tested by variation of the concentration (0.5–1.6 mM) in the presence of 2.5 mM linoleic acid at pH 7 and 37 °C. For kinetic studies, the piperine cleavage was performed with 1.6 mM piperine and 25 mM linoleic acid at pH 7 and 37 °C with varying incubation time (0–48 h). Furthermore, varying $LOX_{Psa}1$ concentrations (10-1000 nkat/mL, 0.6–60 U/mL, 0.015–1.5 mg/mL) were tested (1.6 mM piperine, 25 mM linoleic acid, pH 7, RT, 16 h).

Analysis of the biotransformation products was performed *via* GC after stir bar sorptive extraction (biotransformation in the presence or absence of linoleic acid or $MnSO_4$, and differing enzyme concentrations; see Supporting Information, Section 1.7.1) or after liquid extraction (analysis with different concentrations of linoleic, α-linolenic acid, and piperine, pH and temperature optimum, and kinetic studies; see Supporting Information, Section 1.7.2).

1.5.3 Biotransformation of other aryl alkenes

Biotransformation of the alkenes *trans*-anethole, methyl isoeugenol, and α-methylstyrene (6.7 mM each) to *p*-anisaldehyde, veratraldehyde, and acetophenone was tested at pH 7 and RT using 2.5 mM linoleic acid and 100 nkat/mL (6 U/mL) $LOX_{Psa}1$ (16 h of incubation). Analysis of the reaction products was performed *via* GC after liquid extraction (see Supporting Information, Section 1.7.3).

1.6 HPLC analysis for piperine quantification

After biotransformation, the residual piperine concentration was determined by HPLC. For this, 1 mL acetonitrile was added to the biotransformation samples. The samples were mixed, filtered (syringe filter Chromafil® RC-45/25, Macherey-Nagel, Düren, Germany), and analyzed by a LC-10 system (Shimadzu Deutschland GmbH, Duisberg, Germany) equipped with a reversed phase column (Chromolith Performance RP-18e, 100 × 4.6 mm, Merck) and a UV/VIS detector (SPD-10A VP, Shimadzu Deutschland GmbH). The following gradient was used at ambient temperature: initial 90% solvent A (0.1% formic acid in water) and 10% solvent B (acetonitrile), 10–58% solvent B in 6 min, 58–100% solvent B in 2 min (hold for 1 min), 100–10% solvent B in 4 min. Finally, the system was re-equilibrated with 10% solvent B for 2 min.

10 µL sample were injected and the flow rate was 1.5 mL/min. Piperine was detected at 345 nm and its concentration was quantified from the corresponding peak area using external calibration.

1.7 GC analysis

1.7.1 Quantification of piperonal and 3,4-methylenedioxycinnamaldehyde after stir bar sorptive extraction

Detection and quantification of piperonal, 3,4-methylenedioxycinnamaldehyde, and linoleic acid was performed by GC analysis after stir bar sorptive extraction using Twisters (10 × 0.5 mm, Gerstel, Mülheim, Germany) coated with polydimethylsiloxane. 500 µL sample were diluted with 1.5 mL deionized water, containing 2.5 µg/mL (18 µM) 1,2-dimethoxybenzene as internal standard (IS), and extracted for 1 h under vigorous stirring at 290 rpm. Afterwards, the Twister was rinsed with deionized water, dried with a lint free cloth, and analyzed by gas chromatography. The stir bar was measured *via* TDS3 (thermodesorption system)-GC-FID (flame ionization detector) (Agilent 6890N, Agilent Technologies, Santa Clara, CA, USA) equipped with a cold injection system (CIS 4, Gerstel) and FID detector (250 °C, H_2-flow 35 mL/min, air-flow: 300 mL/min, N_2-flow: 25 mL/min). The initial temperature of the TDS 3 was 20 °C. The sample was heated with 60 °C/min to 300 °C and held for 3 min for splitless desorption. Volatiles were refocused in the CIS 4 at −10 °C on a liner filled with Tenax TA. The CIS was heated to 300 °C with 12 °C/s and held at maximal temperature for 3 min. The sample was injected in the solvent vent mode. As a stationary phase, HP-5 (30 m, 0.32 mm, 0.25 µm, Agilent Technologies) was used. All analyses were performed with a volumetric flow rate of 1.3 mL/min hydrogen using the following temperature program: 40 °C (3 min), a temperature increase of 3 °C per minute until 150 °C, a further temperature increase of 20 °C per minute until 300 °C and a final hold time of 10 min. Piperonal and 3,4-methylenedioxycinnamaldehyde were semi-quantified according to the area of the IS. Biotransformation products were identified using standards and comparison of retention indices with literature. Furthermore, GC mass spectrometry was used for identification (Agilent 6890 N, equipped with a VF-WAX ms column (30 m × 0.25 mm, 0.25 µm, Agilent) and CIS 4; coupled with mass spectrometry (Agilent 5977A) and olfactory detection port (ODP3, Gerstel, 250 °C)). GC–MS analyses were performed with helium as carrier gas, 0.25 µL injection volume and a flow rate of 1.0 mL/min. MS scans were run in a range of m/z 34–500 with a scan rate of 3.1 scans/s. The thermodesorption and GC temperature program was the same as in the GC-FID analyses. Sensory evaluation of 3,4-methylenedioxycinnamaldehyde was performed *via* the ODP3.

1.7.2 Quantification of piperonal and 3,4-methylenedioxycinnamaldehyde after liquid extraction

The biotransformation products were extracted by liquid extraction using 1 mL diethylether containing 10 mg/L (0.1 mM) cyclohexanol (IS). The organic phase was dried with anhydrous sodium sulfate and subsequently analyzed by GC with an Agilent 7890 instrument equipped with a HP-5 column (30 m × 0.32 mm, 0.25 µm, Agilent), an on column injection port and FID. Hydrogen was used as carrier gas at a constant flow rate of 2.1 mL/min. One µL sample was injected *via* an autosampler and measured using the following method: 40 °C (3 min), a temperature increase of 5° C per minute until 230 °C, a further temperature increase of 25 °C per minute until 325 °C and a final hold time of 10 min. The biotransformation products were semi-quantified in relation to the area of the IS.

1.7.3 Quantification of *p*-anisaldehyde, veratraldehyde, and acetophenone

The conversion products were extracted with 1 mL hexane containing 100 mg/L (1 mM) cyclohexanol (IS). The organic phase was dried with anhydrous sodium sulfate and subsequently analyzed by GC. GC measurements were performed with an Agilent 7890 instrument equipped with a DB-WAX UI column (30 m × 0.32 mm, 0.25 µm, Agilent), a split/splitless injector port (1:5) and a FID. Hydrogen was used as carrier gas at a constant flow rate of 2.1 mL/min. One µL sample was injected *via* an autosampler and measured using the following method: 40 °C (3 min), a temperature increase of 10 °C per minute until 230 °C and a final hold time of 10 min. The biotransformation products (*p*-anisaldehyde, veratraldehyde, and acetophenone) were semi-quantified according to the area of the internal standard. Biotransformation products were identified using standards and comparison of retention indices with literature.

2. Results

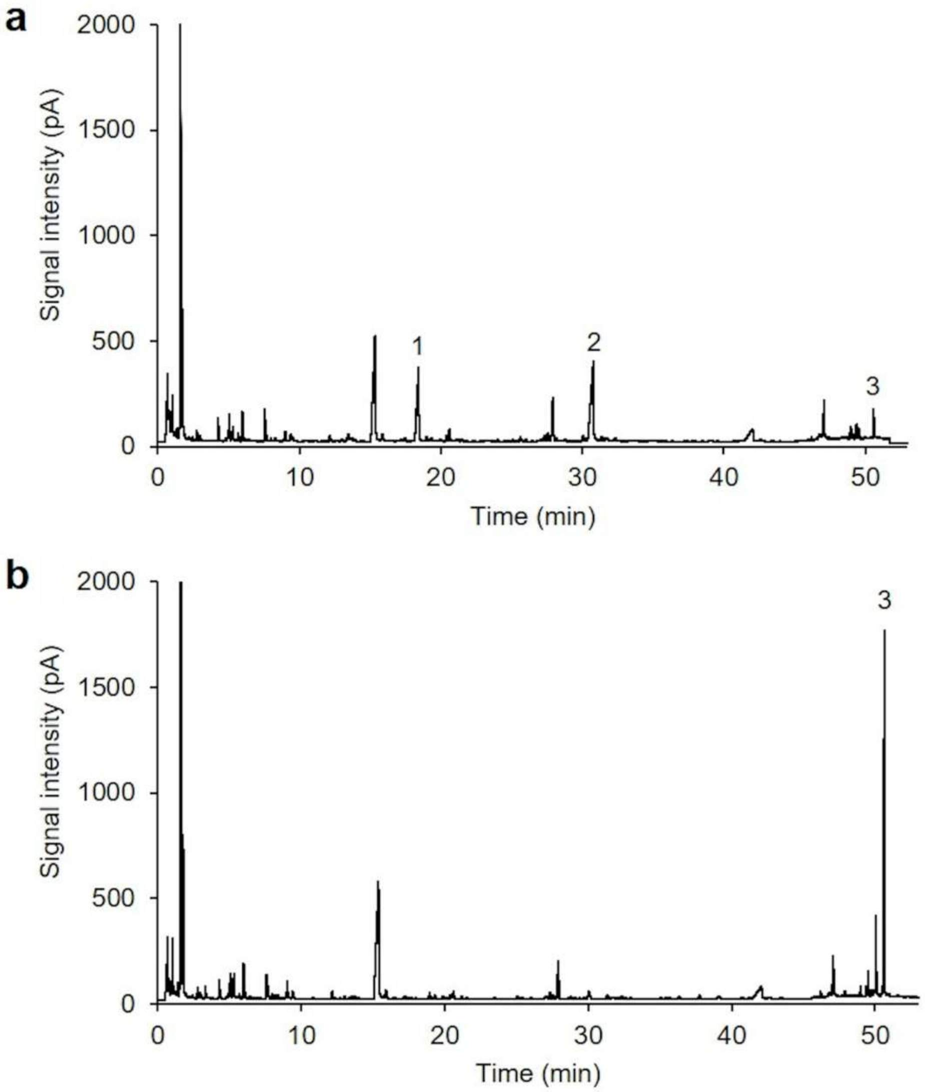

Figure S1. TDS-GC-FID chromatogram of the bioconversion of piperine with lyophilized mycelium of *P. sapidus* after 16 h of incubation. **a**) Active mycelium. **b**) Blank sample with heat inactivated mycelium (4 h, 95 °C). 1: piperonal, 2: 3,4-methylenedioxy-cinnamaldehyde, 3: piperine.

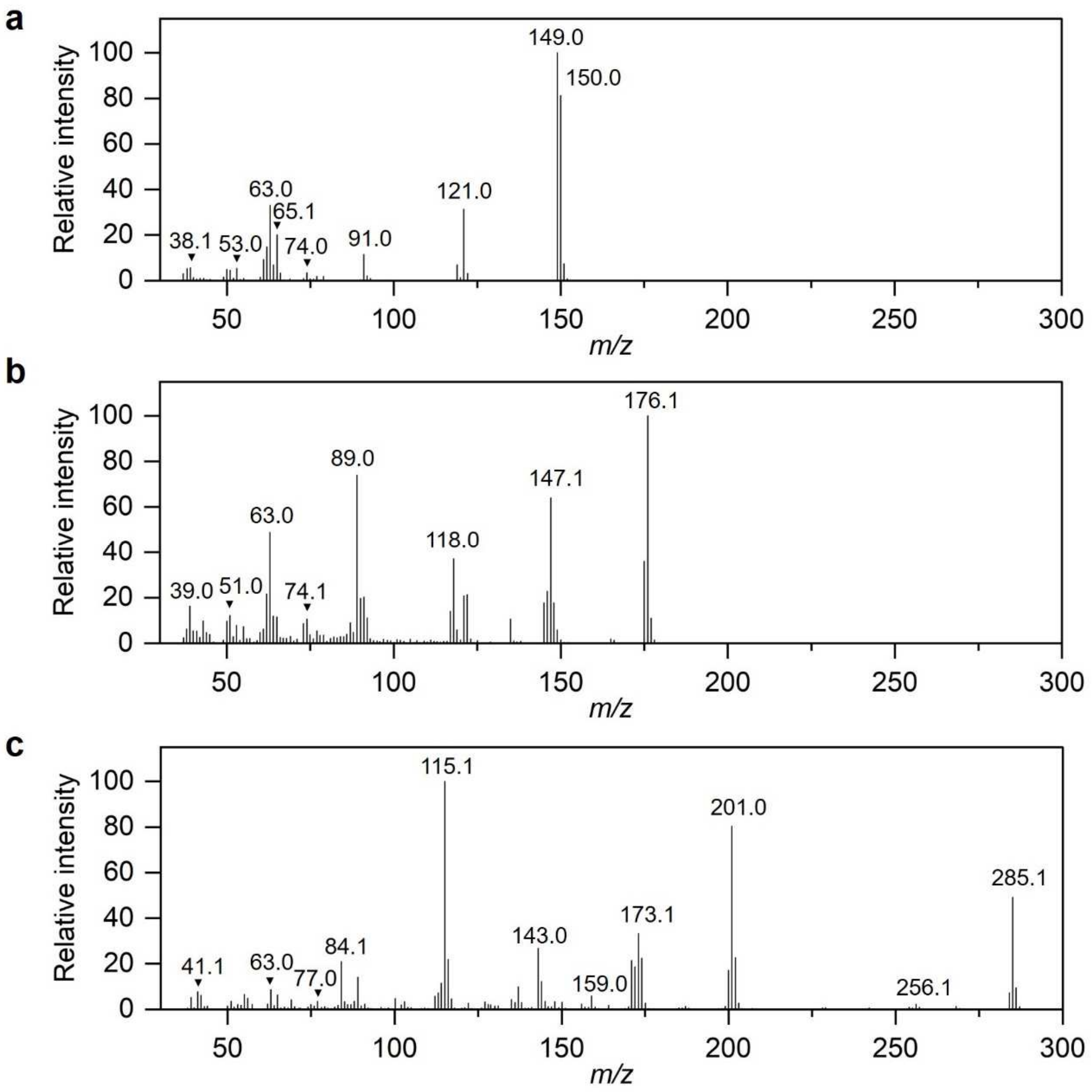

Figure S2. MS spectra resulting from GC-MS analysis that were used for the allocation of the substrate and products to the respective peaks in the TDS-GC-FID chromatogram (Figure S1). **a**) Piperonal, **b**) 3,4-methylenedioxycinnamaldehyde, and **c**) piperine.

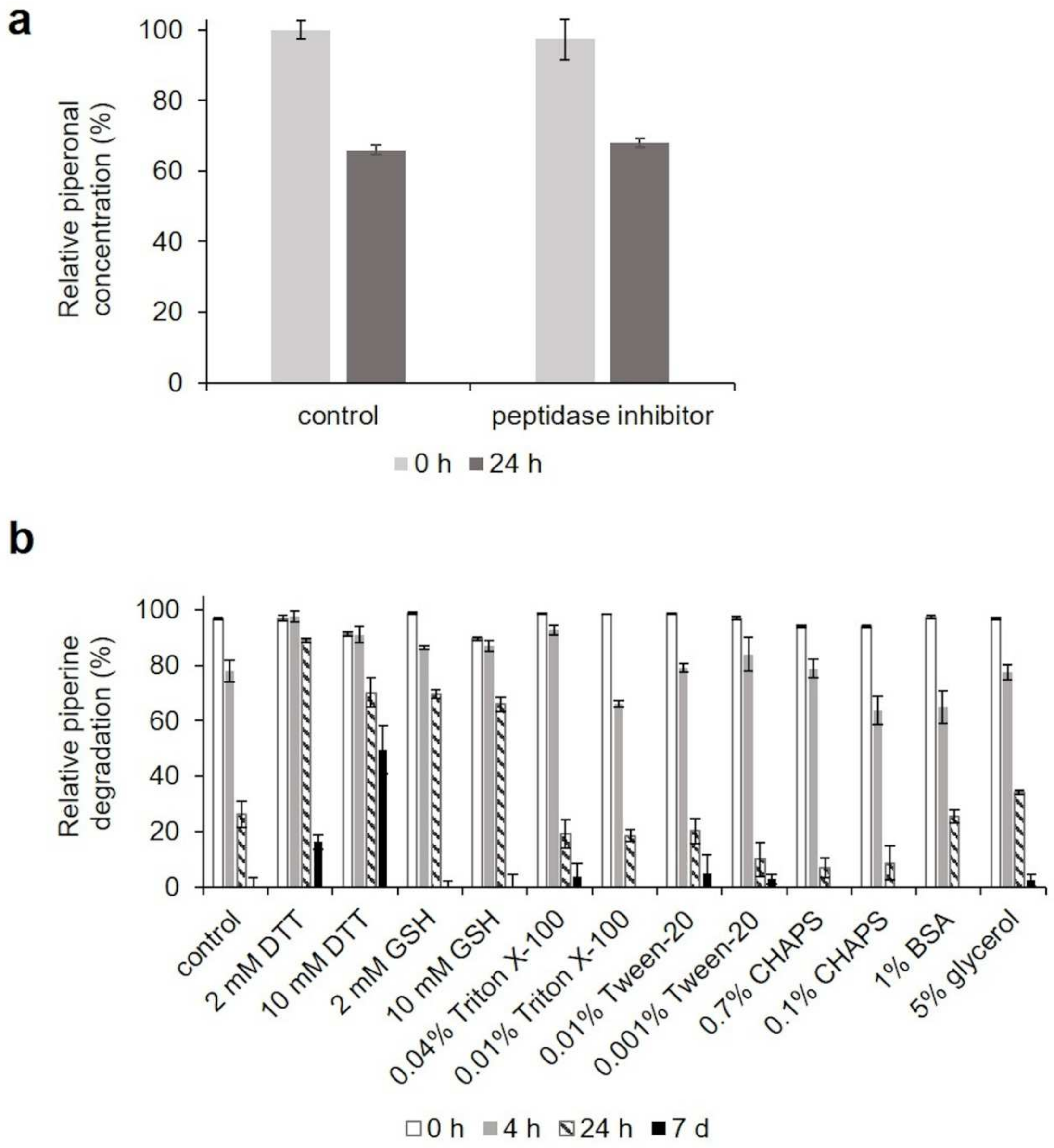

Figure S3. Storage stability of the piperine cleaving enzyme in the soluble fraction after rehydration of the *P. sapidus* mycelium. **a**) Relative piperonal concentration for the biotransformation after storage at 4 °C over 0 and 24 h in the presence or absence of a peptidase inhibitor mix. Concentrations were calculated relative to the value obtained for the control sample after 0 h storage. **b**) Relative piperine degradation for the biotransformation after storage at 4 °C over 0, 4, 24 h, and 7 d in the presence of different stabilizing agents. The degradation was calculated relative to the starting piperine concentration. Control: without addition of stabilizing agent; DTT: dithiothreitol, GSH: glutathione; CHAPS: 3-[(3-cholamidopropyl)dimethylammonio]-1-propanesulfonate; BSA: bovine serum albumine. For the detergents (Triton X-100, Tween-20, and CHAPS) one concentration above and one below the critical micellar concentrations were used.

Table S1. Best hits resulting from the protein sequencing of protein band 1 to 6 from the SDS-PAGE (Figure 1e).

Protein band	Protein (GenBank accession no.) and amino acid sequence
1	Glycoside hydrolase (belonging to family 3) of *Pleurotus ostreatus* PC15 (KDQ23735.1)<br>MNGLCVGNIPPVQNWPGLCLEDSPLGVRFGDFSTAFPTAINAAATWNRRLIRLRGLFMGQEHVGKGVNVALG<br>PMMNMGRVANGGRNWEGFGADPFFAGEAAYETILGMQEAGVQACAKHFINNEQEHKRTESTSDVDDRTQHEI<br>YAHPFLKSVMAGVASVMCSYNQINGTFACENDKMLNDVLKREFGFQGYVMSDWQATHSTHSANDGLDMTMPG<br>DITFNSGDSWFGGNLTTSVRDNQTPEARLDDMATRIIAAWYLLKQQRSDFPTPNFDAFRPDNEQTNFHIDVQ<br>DDHGDLVREMGAASTVLLKNVRGALPLRKPRSLVLVGSDAGPGVIGPNHFSDQGGVDGVLAMGWGSGTANFT<br>YLVSPYEAISARARKDHTTLSWIFDDFNLARAGNMAIGRSAALVFLNSDSGEGYITVDGNEGDRRNLTAWHG<br>GDNLVTAVAAQNNNTIVVVHSVGPLILEPWIEHPNVTAVVWAGVSGTETGNALVDILYGAWNPSGRLPYTIA<br>KRPEDYPAQLVLGGGGAENIIPIPYTEGLEIDYRHFDAKNITPRFEFGFGLSYTTFEYSNLKVSKIDSPDHV<br>QSDLERAWAAGKASPHGQGSSTALYLHRPAFRVTFDVKNTGKLFGGDIPQLYVNMPASSGEPPSILKGFTNI<br>ELSPNERRTVTINLSRYDLSIWDTAAQGWAKPAGRIAITVGASSRDARLHGRIPL
2, 3*, 4*, 5*, 6*	Lipoxygenase (LOX$_{Psa}$1) of *Pleurotus sapidus* (CCV01581.1)<br>MVHNISLSSRKALHNVHLPYMVQLPKPTGYNVALKNAAEGYDKARRMVAWLYDIADYESSIPQTFTLQQKTD<br>KYTWELSDNFPPHLAVVPPDQSVSAPSIFSPVRLAQTLLIMSSLWYDDHTDLAPGPEQNTMQKLTQWNQERH<br>KDQGWLIKDMFNAPNIGLRNDWYTDEVFAQQFFTGPNSTTITLASDVWLTAFTSEAKAQGKDKVIALFESAP<br>PNSFYVQDFSDFRRRMGAKPDEELFNDSDGAMRYGCAAVALFYLTAMGKLHPLAIIPDYKGSMAASVTIFNK<br>RTNPLDISVNQANDWPWRYAKTCVLSSDWALHEMIIHLNNTHLVEEAVIVAAQRKLSPSHIVFRLLEPHWVV<br>TLSLNALARSVLIPEVIVPIAGFSAPHIFQFIRESFTNFDWKSLYVPADLESRGFPVDQLNSPKFHNYAYAR<br>DINDMWTTLKKFVSSVLQDAQYYPDDASVAGDTQIQAWCDEMRSGMGAGMTNFPESITTVDDLVNMVTMCIH<br>IAAPQHTAVNYLQQYYQTFVPNKPSALFSPLPTSIAQLQKYTESDLMAALPLNAKRQWLLMAQIPYLLSMQV<br>QEDENIVTYAANASTDKDPIIASAGRQLAADLKKLAAVFLVNSAQLDDQNTPYDVLAPEQLANAIVI
4	Alkene cleaving DyP-type peroxidase (PsaPOX) of *Pleurotus sapidus* (QIV15482.1)<br>MTTPAPPLDLNNIQGDILGGLPKKTETYFFFDVTNVDRFKANMTQFIPHVKTSAGIVKDREAIKEHKRQKRP<br>GLVPMAAVNVSFSHLGLQKLGITDDLSDSSFTTGQRKDAEVLGDPGTKNGDTFTPAWEAPFLKDIHGVIFVA<br>GDCHASVHKKLDEIKHIFGVGTSHASISEVTHVRGDVRPGQVSAHEHFGFLDGISNPAVDQFDQNPFPGQDS<br>IRPGFILAKENGDSRAAARPDWAKDGSFLTFRYLFQMVPEFDDFLESNPIVLPGLSRKEGSELLGARIVGRW<br>KSGAPIEITPLKDDPKLGADAQRNNNFDFGDSLVRGDQTKCPFAAHIRKTYPRNDLEGPPLNADIDNRRIIR<br>RGIQFGPEVTSQEHHDKKTHHGRGLLFVCYSSSIDDGFHFIQQSWANAPNFPVNAVTSAGPIPPLDGVIPGF<br>DAIIGQKVGGGIRQISGTNPNDPTTNITLPDQDFVIPRGGEYFFSPSISALKTKFAAGVASSAPQAQAPIST

Peptides identified by protein sequencing are underlined. * Peptidolytic degradation of LOX$_{Psa}$1 resulted in multiple protein bands as described by Plagemann *et al.*[12]

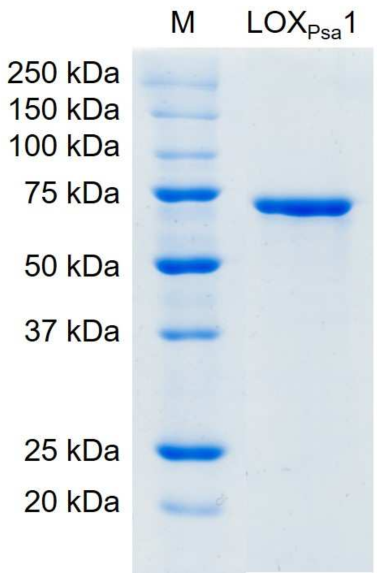

Figure S4. SDS-PAGE analysis of the purified recombinant LOX$_{Psa}$1. M: molecular mass marker, LOX$_{Psa}$1: purified enzyme.

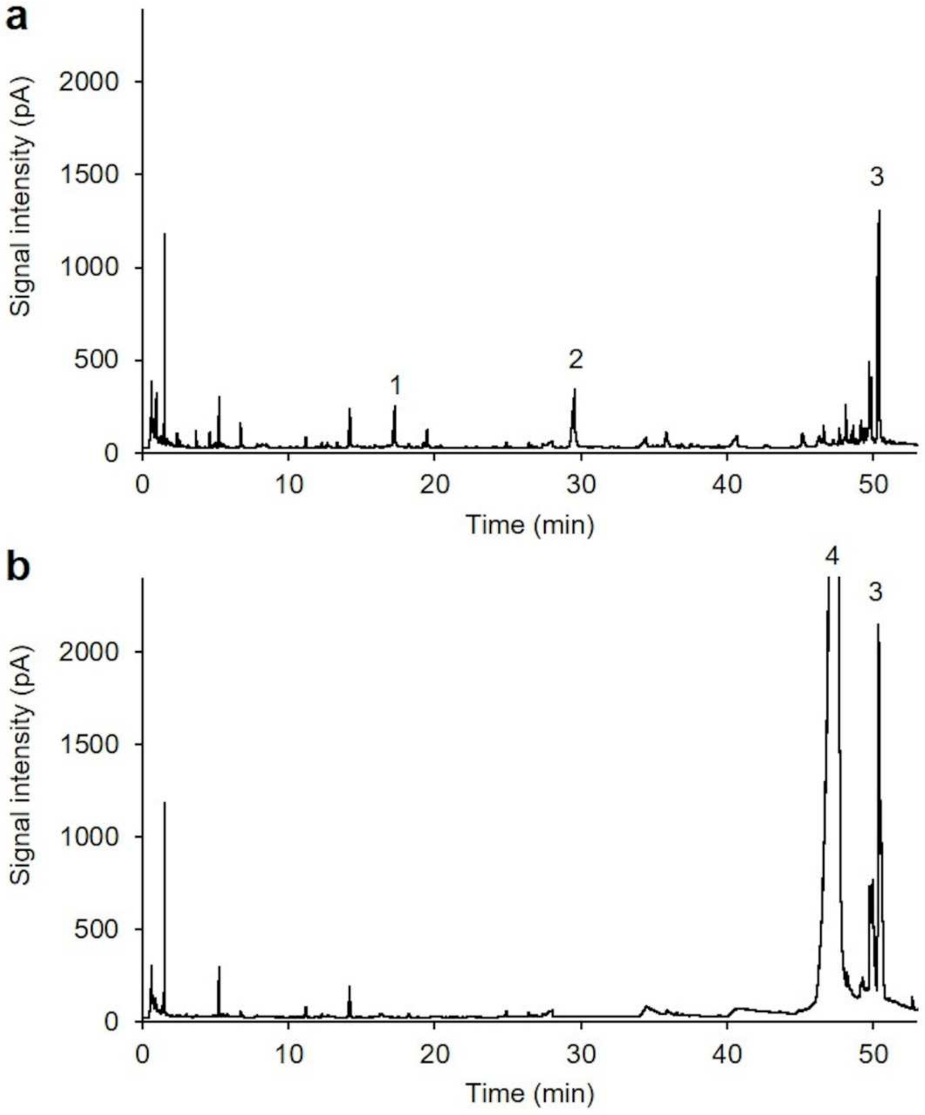

Figure S5. TDS-GC-FID chromatogram of the bioconversion of piperine with LOX_{Psa}1 after 16 h of incubation. **a**) Recombinant LOX_{Psa}1 (100 nkat/mL, 6 U/mL). **b**) Blank sample with heat inactivated enzyme (1 h, 95 °C). 1: piperonal, 2: 3,4-methylenedioxy-cinnamaldehyde, 3: piperine, and 4: linoleic acid.

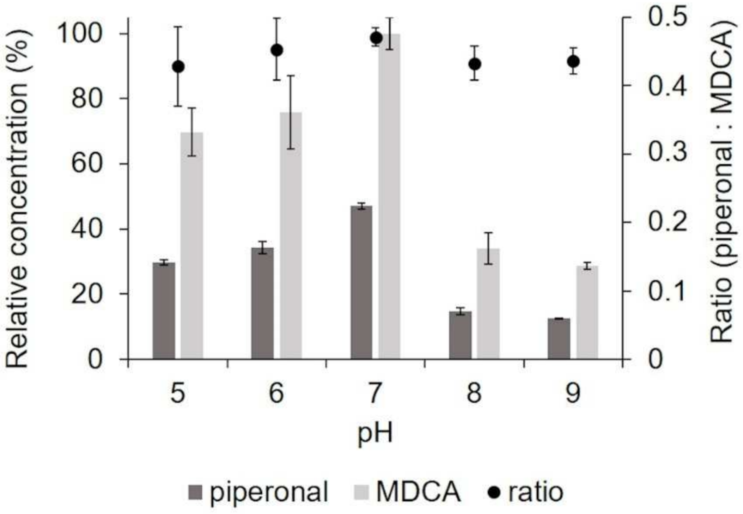

Figure S6. pH optimum of the piperine biotransformation using 100 nkat/mL (6 U/mL) $LOX_{Psa}1$ in the presence of 2.5 mM linoleic acid and 1 mM piperine at RT after 16 h. Concentrations are relative to the highest product concentration. MDCA: 3,4-methylenedioxycinnamaldehyde.

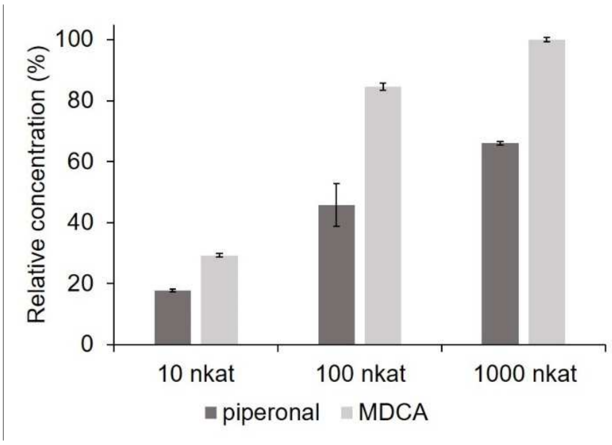

Figure S7. Piperine biotransformation with different $LOX_{Psa}1$ activities in the presence of 2.5 mM linoleic acid and 1 mM piperine at pH 7 and RT after 16 h. Concentrations are relative to the highest product concentration. MDCA: 3,4-methylenedioxycinnamaldehyde.

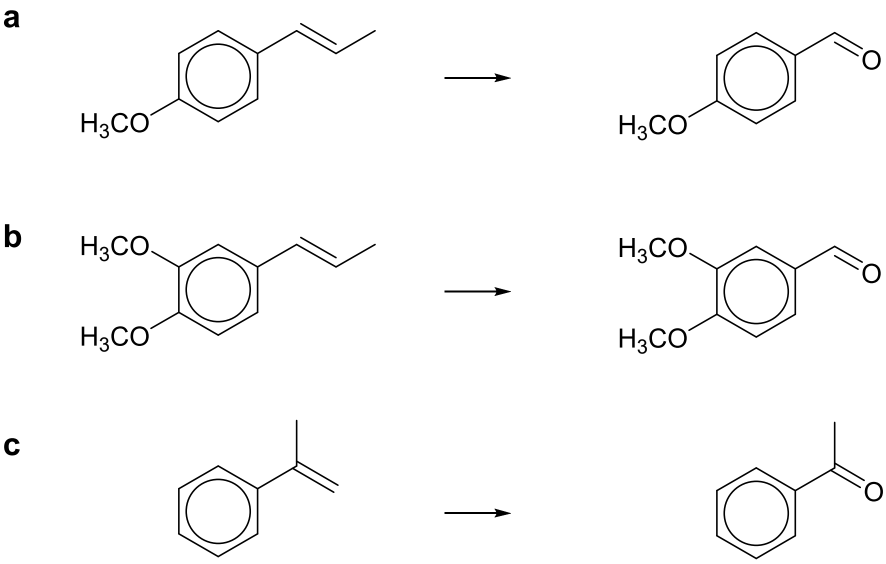

Scheme S1. Bioconversion of **a**) *trans*-anethole to *p*-anisaldehyde, **b**) (*E*)-methyl isoeugenol to veratraldehyde, and **c**) α-methylstyrene to acetophenone by $LOX_{Psa}1$.

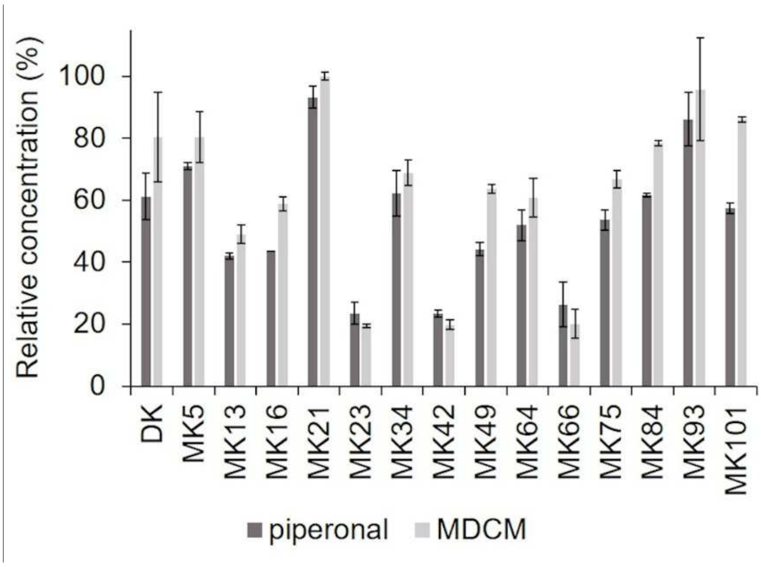

Figure S8. Piperine biotransformation with different *P. sapidus* strains in the presence of 2.5 mM linoleic acid and 1 mM piperine at pH 7 and RT after 16 h. DK: parental strain used in the presented study; MK: monokaryotic daughter strains. Concentrations are relative to the highest product concentration. MDCA: 3,4-methylenedioxycinnamaldehyde.

3. References

[1] R. Gallagher, R. Shimmon, A. M. McDonagh, *Forensic Sci. Int.* **2012**, *223*, 306–313.

[2] N.-K. Krahe, R. G. Berger, M. Witt, H. Zorn, A. B. Omarini, F. Ersoy, *Int. J. Mol. Sci.* **2021**, *22*, 1363.

[3] D. Linke, R. Leonhardt, N. Eisele, L. M. Petersen, S. Riemer, M. Nimtz, R. G. Berger, *Bioprocess Biosyst. Eng.* **2015**, *38*, 1191–1199.

[4] U. Krings, N. Lehnert, M. A. Fraatz, B. Hardebusch, H. Zorn, R. G. Berger, *J. Agric. Food Chem.* **2009**, *57*, 9944–9950.

[5] D. Linke, A. B. Omarini, M. Takenberg, S. Kelle, R. G. Berger, *Appl. Biochem. Biotechnol.* **2019**, *187*, 894–912.

[6] N.-K. Krahe, R. G. Berger, F. Ersoy, *Molecules* **2020**, *25*, 1536.

[7] K. Zelena, U. Krings, R. G. Berger, *Bioresour. Technol.* **2012**, *108*, 231–239.

[8] R. H. Leonhardt, I. Plagemann, D. Linke, K. Zelena, R. G. Berger, *J. Mol. Catal. B Enzym.* **2013**, *97*, 189–195.

[9] M. M. Bradford, *Anal. Biochem.* **1976**, *72*, 248–254.

[10] I. Plagemann, K. Zelena, P. Arendt, P. D. Ringel, U. Krings, R. G. Berger, *J. Mol. Catal. B Enzym.* **2013**, *87*, 99–104.

[11] H. T. S. Britton, R. A. Robinson, *J. Chem. Soc.* **1931**, 1456–1462.

[12] I. Plagemann, U. Krings, R. G. Berger, *Zeitschrift für Naturforsch. C* **2014**, *69*, 149–154.
